# Quacks and Quackery

**Published:** 1869-02

**Authors:** 


QUACKS AND QUACKERY.—It may be expected from what I have already said, that I shall forthwith proceed to call in question the consort of the people with quacks; or pass an anathema on quacks and quackery individually and collectively. From this I abstain. Consort of the community with quacks is so obviously the result of ignorance, that if the most moderate share of attention were given to the subject, if a tithe of the attention I have prayed on behalf of the profession itself were given to the subject, commun ion with quacks and their foolish arts would naturally cease. As to the quacks, to notice them were to elevate them. Belonging strictly to the worst of the criminal classes, they are moved by no sentiments which the most acute criticisms could touch. A professed gambler may have sense of honor, a pickpocket may have skill, a professed burglar may have courage; the professed quack has the sins of them all, the saving qualities of none. He is, because he is permitted, a forced necessity of morbid minds. One thing only would I note in his history as most wonderful, viz.: that the grand disseminators of human knowledge, the grand teachers of moral truths, the proprietors of the fourth estate allow him unblushingly to deface their fair pages with his falsehood, his snares, his open loathsome sin. Day by day the press, in daring faultless language, and sentiment, exposes vice and purifies the thought of the world; day by day, in the greater number of its organs, it sells itself to the advertisement of immoralities worse than the worst it endeavors to remove.—*From Dr. Richardson's address to the St. Andrews Medical Graduates' Association.*—*Western Journal of Medicine.*

